# Asymptomatic Gangrenous Acute Cholecystitis: A Life-Threatening Condition

**DOI:** 10.7759/cureus.36672

**Published:** 2023-03-25

**Authors:** Victor J Medina, Annia M Martial, Tulika Chatterjee

**Affiliations:** 1 Internal Medicine, University of Illinois College of Medicine Peoria, Peoria, USA

**Keywords:** gangrenous cholecystitis, biliary sludge, painless obstructive jaundice, lap cholecystectomy, gall bladder diseases and gallstones

## Abstract

Gangrenous gallbladder (GGB) is a life-threatening complication of acute cholecystitis, which happens due to gallbladder (GB) wall ischemia and necrosis. Delaying the diagnosis of GGB is life-threatening and may happen in asymptomatic patients. We present a case of an elderly male patient with a history of gastric carcinoid tumor, with partial gastric resection, who presented with generalized weakness and jaundice. His total bilirubin was elevated and an ultrasonography and computed tomography (CT) scan of the abdomen showed evidence of acute cholecystitis. An endoscopic retrograde cholangiopancreatography (ERCP) the day after admission showed no evidence of choledocholithiasis or cholangitis. It was during laparoscopic cholecystectomy three days later that the diagnosis of GGB was made as the GB was found to be necrotic with extensive adhesions. The patient also required intravenous antibiotics prior to discharge. This case illustrates risk factors for the development of gangrenous cholecystitis, physical findings in asymptomatic patients, and the importance of early diagnosis in order to reduce morbidity in this patient population.

## Introduction

Acute cholecystitis is a serious and life-threatening condition and the most common complication of cholelithiasis. Interestingly, it may remain asymptomatic in approximately 1-2% of patients [1]. Approximately 2-29.6% of patient cases of acute cholecystitis may progress to gangrenous cholecystitis which occurs due to the gallbladder (GB) wall ischemia and necrosis secondary to GB inflammation and distention, with or without cystic artery thrombosis [1,2]. Gangrenous cholecystitis or gangrenous gallbladder (GGB) is a serious complication of acute cholecystitis that is associated with high morbidity and mortality [1]. Male gender, elevated white blood cell (WBC) count, age greater than 45 years, diabetes, and cardiovascular disease are risk factors for the development of GGB [3]. Diagnosing the condition is challenging, as it is difficult to differentiate from uncomplicated acute cholecystitis based on clinical presentation and laboratory workup. Therefore, frequently the diagnosis is made intra-operatively by direct visualization of the GB [4]. To complicate the diagnostic pathway, some patients, especially the elderly and those with diabetes, may remain asymptomatic even when they develop GGB. Underlying neuropathy is thought to be the cause of the paucity of pain [5]. Notably, surgery should be performed when there is suspicion of acute cholecystitis regardless of the presence or absence of GGB. We present the case of an elderly male patient with a complex medical history that was transferred to us from an outside hospital for fatigue, a low-impact fall, slight jaundice, and abnormal liver function tests.

## Case presentation

An 85-year-old male patient presented from an outside hospital with worsening generalized weakness and slight yellowing of skin ongoing for one week. His past medical history was significant for adenocarcinoma of the prostate, status post laparoscopic prostatectomy, carcinoid tumor of the stomach, status post partial resection and continued treatment with octreotide, pernicious anemia likely secondary to partial carcinoid tumor resection, and hypothyroidism. The patient denied any recent illness, fever, chills, nausea, vomiting, or abdominal pain. The patient also denied tobacco use but did endorse a long history of three to four alcoholic beverages daily. On arrival at our facility, the patient was hemodynamically stable and his vitals were all within normal limits. On examination, he was icteric, non-tender to palpation of the abdomen, and had a positive Murphy’s sign. Our suspicion of biliary disease, specifically acute cholecystitis, led us to begin the appropriate workup and management.

**Figure 1:**
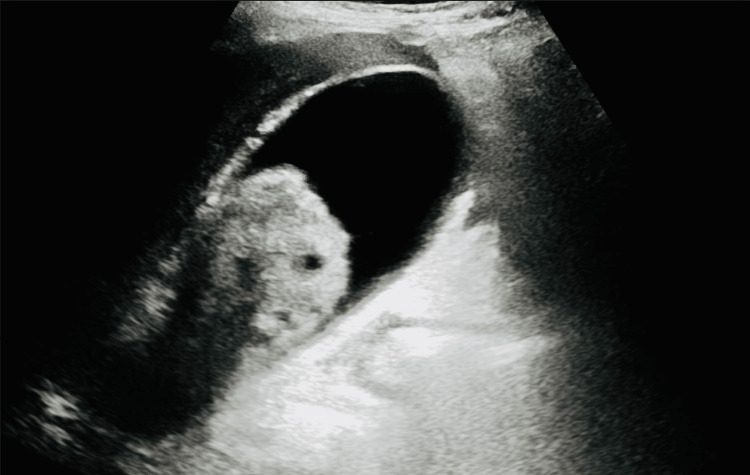
Gallbladder sludge ball

**Figure 2:**
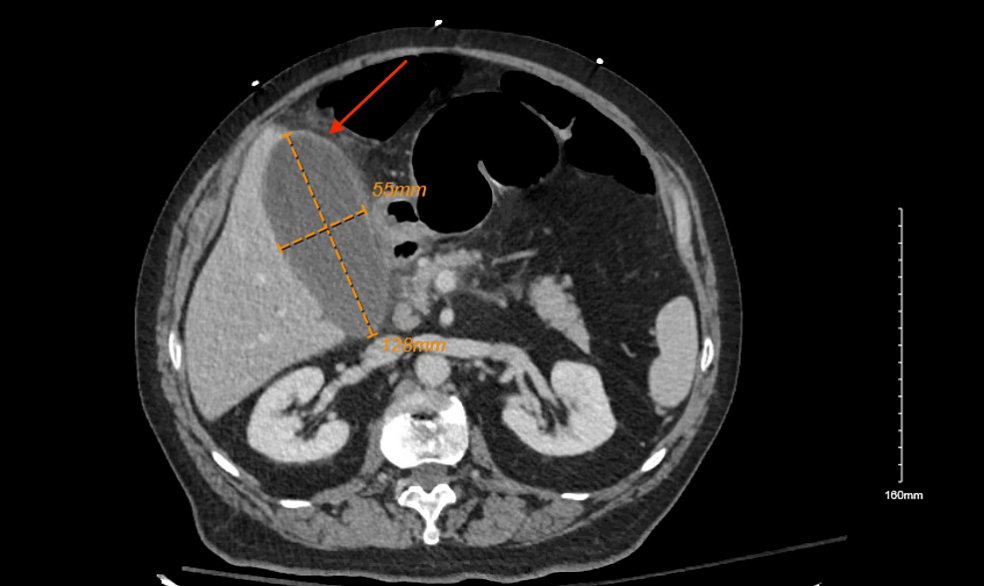
CT of the abdomen and pelvis demonstrating the size of the gallbladder and the red arrow indicating stranding surrounding the gallbladder CT: computed tomography

Laboratory workup revealed an elevated WBC count with 89.4% neutrophil predominance, total bilirubin, aspartate transaminase (AST), alanine aminotransferase (ALT), and alkaline phosphatase (Table 1). Other labs including lipase, COVID-19, influenza A and B, lactic acid, and blood cultures drawn at the outside hospital, and repeat cultures at our facility were unremarkable. An ultrasound of the abdomen showed a 6.5 x 3 cm echogenic sludge ball in the GB (Figure 1), a normal common bile duct (CBD) diameter of 0.5 centimeters, and a negative sonographic Murphy’s sign. A computed tomography (CT) scan of the abdomen showed GB distention, wall thickening, and stranding (Figure 2). An endoscopic retrograde cholangiopancreatography (ERCP) the day after admission showed a non-dilated CBD with no filling defects. Overall, ERCP did not show any evidence of cholangitis or CBD obstruction, but a small amount of sludge was removed from CBD and a stent was placed.

**Table 1:** Demonstrates the patient's abnormal blood work along with the normal range of values

Blood Work	Patient Test Results	Normal Range	Interpretation of the Results
White Blood Cell Count	20.34 x 10^3 micro-liters	4.5 to 11 x 10^3 micro-liters	Elevated
Total Bilirubin	8.6 mg/dL	0.1 to 1.2 mg/dL	Elevated
Direct Bilirubin	7.5 mg/dL	0.0 to 0.5 mg/dL	Elevated
Aspartate Transaminase (AST)	104 U/L	8 to 33 U/L	Elevated
Alanine Aminotransferase (ALT)	275 U/L	4 to 36 U/L	Elevated
Alkaline Phosphatase	155 U/L	44 to 147 U/L	Elevated

The following day after the ERCP, the patient underwent laparoscopic cholecystectomy. During the surgery, his GB was found to be highly distended and necrotic with extensive adhesions of the omentum, duodenum, and colon to the GB. On the first attempt to grasp the GB, it tore, releasing large amounts of bile and small, smooth-surfaced brown gallstones with a maximum diameter of 0.4 mm. Extensive blunt dissection of Calot’s triangle was done during the procedure to release the adhesions. After the patient’s cholecystectomy, his liver enzymes began to slowly decline and the patient was given a seven-day course of intravenous piperacillin-tazobactam.

Histopathology of the surgical specimen confirmed the diagnosis of gangrenous cholecystitis. The patient remained stable and was successfully discharged from the hospital. He was noted to be doing well during his follow-up with surgery a month later with normalization of hepatic enzymes.

## Discussion

GGB is a late complication of acute cholecystitis. It happens in the late stages of GB inflammation due to wall ischemia, necrosis, and perforation [2,6]. According to a study by Thomsen et al., acute cholecystitis tends to be more common in patients with a history of malignancy, such as our patient [7]. Distention of the GB leads to increased wall pressure and eventual necrosis of the GB wall, which can be associated with cystic artery thrombosis [8,9]. The clinical presentation and laboratory findings of GGB can be indistinguishable from uncomplicated acute cholecystitis and thus diagnosis can be delayed leading to increased morbidity and mortality [2,10]. The correct diagnosis of GGB is often made intra-operatively [8], as was the case with our patient where extensive laboratory workup, ultrasound, CT of the abdomen, and even ERCP did not diagnose GGB prior to surgery.

Usual symptoms of acute cholecystitis including right upper quadrant (RUQ) abdominal pain, radiation to the shoulder, nausea, and vomiting may be absent in elderly patients, who may even have atypical symptoms. For instance, unusual symptoms like intractable hiccups have been reported as the only presenting symptoms of GGB in elderly males [11]. In another instance, a 71-year-old male was found to have a perforated GGB on CT imaging [12].

Advanced age, cardiovascular disease, and diabetes have been identified as risk factors for GGB [2,6]. Patients with long-standing diabetes can be completely asymptomatic of GGB due to neuropathy leading to the inability to appreciate pain. Other cases have reported patients with diabetes that were diagnosed with GGB either on imaging or intraoperatively, while the initial abdominal exam was benign [5,4,13,14]. The patient that we presented in this report did not have a history of diabetes but he did have partial resection of the stomach for a gastric carcinoid tumor, which could have led to splanchnic nerve damage. Another presentation of GGB reported that a patient presented with RUQ pain and then the pain resolved, but CT imaging showed discontinuous GB wall and pericholecystic fluid pockets, demonstrating perforated GGB [12]. Thus, perforation of a distended GB may lead to the resolution of pain leading to a false impression of clinical improvement.

Furthermore, elevated WBC count, elevated C reactive protein, and male gender have been identified as other risk factors for developing GGB. Merrium et al. reported that WBC counts greater than 17,000/mm^3^ was a predictor for the development of GGB [15]. Ages greater than 45 years, male gender, and ultrasound findings of a negative Murphy’s sign have been reported as predictive factors for GGB [3]. A negative Murphy’s sign due to extensive denervation from necrosis should raise clinical suspicion for GGB [3]. A delay in presentation to the hospital from the time of symptom onset has also been associated with the risk of developing GGB [2].

Although GGB can be a difficult diagnosis to make, CT with contrast has been found to have sensitivity up to 96% in the diagnosis of GGB with the highest specificity for gas in GB wall or lumen, intraluminal membranes, irregular or absent GB wall, and pericholecystic abscess [8]. CT and ultrasound imaging might be indicative of GGB but may not be 100% reliable in ruling out GGB, as was the case with our patient. In our patient, the CT showed only a thickened GB wall and there was no element leading toward GGB. It was only intra-operatively that diagnosis of GGB was made by direct visualization and confirmed by histopathology. The literature reports that between 8% and 75% of these patients require conversion of laparoscopic procedure to open cholecystectomy [8,15]. Fortunately, our patient was successfully treated via laparoscopic cholecystectomy and subsequent Jackson-Pratt drain placement.

GGB has been associated with almost 22% mortality and a complication rate as high as 25%. Untreated GGB can lead to perforation, peritonitis, and abscess formation [11,4,13]. The clinical presentation of GGB can be varied and deceptive. It is imperative for clinicians to be cognizant of the clinical entity of GGB and the risk factors associated with its development.

## Conclusions

GGB is a late complication of acute cholecystitis that may present asymptomatic due to extensive necrosis leading to denervation. Jaundice is caused by severe inflammation of the GB that can lead to distention and compression of the bile ducts and may be the only presenting physical finding. It is important that clinicians are aware of some of the risk factors for developing gangrenous cholecystitis, such as male gender, age, and patients with diabetes or other comorbid conditions. Failure to recognize physical findings and risk factors can lead to delayed diagnosis, which is associated with increased morbidity and mortality in patients with GGB. We hope that this case serves as a reminder or learning opportunity of the different GB disease processes and how the delay in the diagnosis in asymptomatic patients may lead to detrimental patient outcomes.
